# Septins as membrane influencers: direct play or in association with other cytoskeleton partners

**DOI:** 10.3389/fcell.2023.1112319

**Published:** 2023-02-17

**Authors:** Béatrice Benoit, Christian Poüs, Anita Baillet

**Affiliations:** ^1^ INSERM UMR-S 1193, UFR de Pharmacie, University Paris-Saclay, Orsay, France; ^2^ Laboratoire de Biochimie-Hormonologie, Hôpital Antoine Béclère, AP-HP, Hôpitaux Universitaires Paris-Saclay, Clamart, France

**Keywords:** septin, membrane, cytoskeleton, actin, microtubule, intermediate filament, ESCRT

## Abstract

The cytoskeleton comprises three polymerizing structures that have been studied for a long time, actin microfilaments, microtubules and intermediate filaments, plus more recently investigated dynamic assemblies like septins or the endocytic-sorting complex required for transport (ESCRT) complex. These filament-forming proteins control several cell functions through crosstalks with each other and with membranes. In this review, we report recent works that address how septins bind to membranes, and influence their shaping, organization, properties and functions, either by binding to them directly or indirectly through other cytoskeleton elements.

## Introduction

**FIGURE 1:**
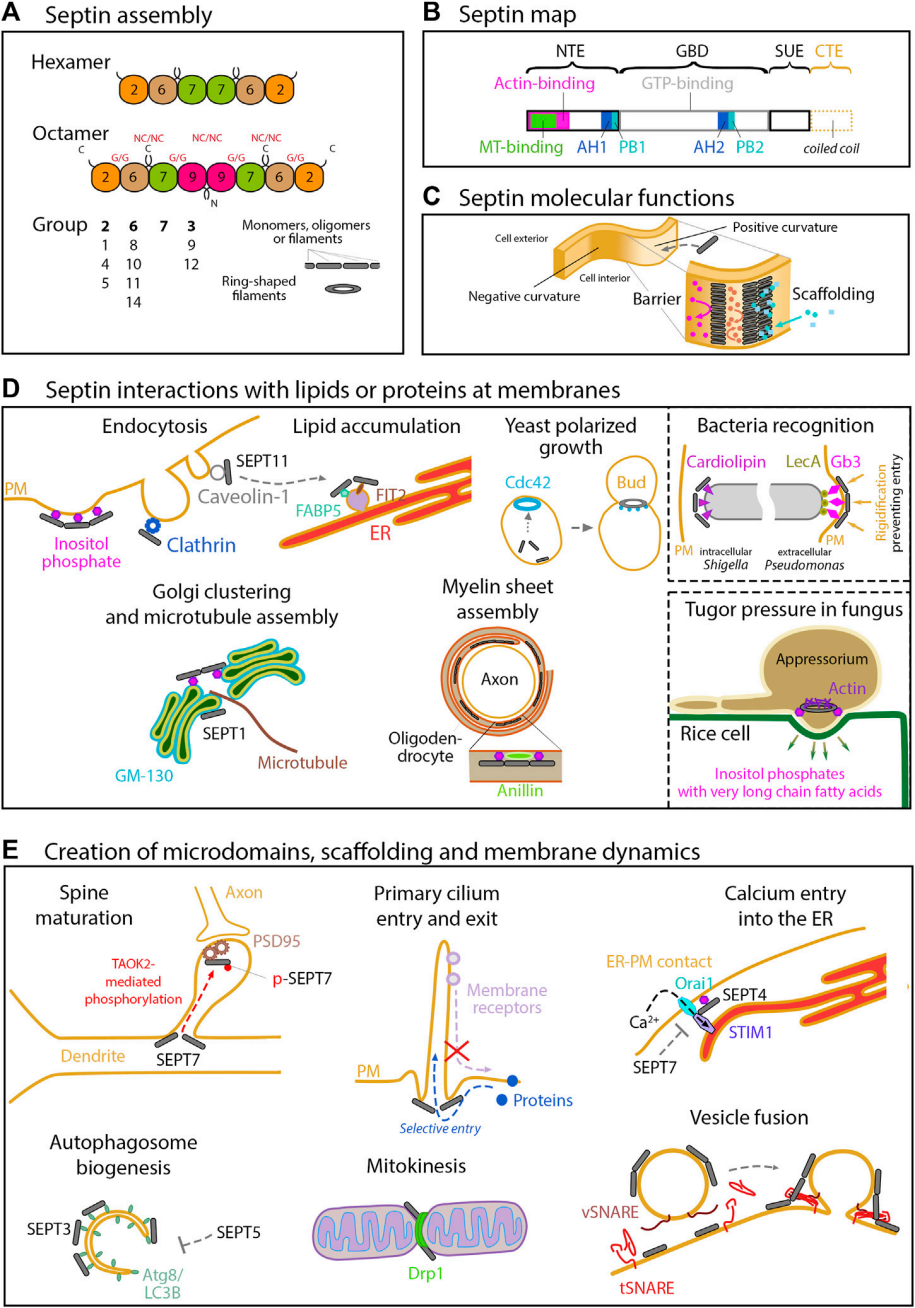
Septin structure, assembly and functions related to membranes. **(A)** Assembly of the septin cytoskeleton. In humans, the septin family comprises 13 members organized into 4 groups (2, 6, 7 and 3) according to their sequence homology. The organization of the four septin group into hexamers and octamers is depicted. Septin monomers either interact through their N- and C-terminal domains (NC/NC interfaces), or through their GTP-binding domain (G/G interfaces). These oligomers can then assemble into filaments or into rings. **(B)** Structural domains encountered in septin proteins. All the septins exhibit a GTP-binding domain (GBD), a septin unique element (SUE), and N- and C-terminal extensions (NTE, CTE) of variable length. Note that the CTE, which contains one or more coiled-coil regions is absent in group 3 septins. Membrane binding is mediated by two AH (amphipathic helix) and PB (polybasic domain) motifs. SEPT9 exhibits physical binding to F-actin through the first half of its NTE, and to microtubules through its N-terminal K/R-R/x-x-D/E repeat motifs. **(C)** Septin molecular functions. At the molecular level, septins can form diffusion barriers or act as scaffolds, as illustrated here in the context of binding to a positively curved membrane. As a barrier, septins can either restrict entry or concentrate molecules within a given domain. **(D)** Septins interact with lipids or proteins at membranes. Septins are enriched at positively curved membranes by binding to phosphoinositides. Through binding to other specific lipids (cardiolipin, Gb3) in bacteria, and through binding to very long chain fatty acid-bearing phosphoinositides in M. orizae fungus, septins ultimately regulate pathogen invasion. In yeast, the membrane recruitment of septins follows high concentrations of active Cdc42, directing further outgrowth of the bud. Through binding to specific proteins (Clathrin, Caveolin, FABP5, FSIT2, Anillin or GM130), septins are involved in a large array of cell processes in higher eukaryotes, including endocytosis, lipid storage, myelin sheet assembly, Golgi integrity and function in nucleating microtubules. **(E)** Septins can act as barriers or scaffolds at membranes. SEPT7 phosphorylation mediates PSD95 stability and restricts its mobility to favor dendritic maturation. At the base of primary cilia, they control protein entry and retain signaling receptors. Septins also organize endoplasmic reticulum-plasma membrane junctions for STIM1-Orai1 calcium signaling. Septins are regulators of autophagy, especially in yeast and neurons, where an interaction with Atg8 has been observed. SEPT2 can recruit Drp1 to mitochondrial constriction sites to favor Drp1-mediated fission. Septins also contribute to control vesicular trafficking machinery at several steps, including the SNARE-mediated membrane fusion.

Septins constitute a family of GTPases considered as the fourth element of the cytoskeleton (Mostowy and Cossart, 2012). They are conserved from fungi to animals but are absent from plants (Shuman and Momany, 2022). According to their structure homology, septins are clustered into four classes, among which they are interchangeable (Kinoshita, 2003). Septins assemble into palindromic hetero-oligomers (Sirajuddin et al., 2007) with a subunit order that has recently been revised within human septin hexamers or octamers (McMurray and Thorner, 2019; Mendonça et al., 2019; Soroor et al., 2021) (Figure 1A). These complexes organize into filaments by annealing (Bridges et al., 2014) to form bundles, gauzes or rings depending on the cell context (Cavini et al., 2021; Woods and Gladfelter, 2021). These filaments are non-polar and do not behave as tracks for dedicated motors. However, they are dynamic since they can exchange internal segments and reorganize (Schmidt and Nichols, 2004). Septin filaments often coalign with membranes, actin fibers or in some circumstances with microtubules, and mostly act as diffusion barriers or scaffolds to perform their functions (Spiliotis and McMurray, 2020; Spiliotis and Kesisova, 2021; Spiliotis and Nakos, 2021). As recently suggested for the control of neuronal trafficking, a “septin code” including genetic and post-translational modification diversity might explain the spatiotemporal adaptation to cell types or to local needs (Spiliotis and Nakos, 2021).

**FIGURE 2:**
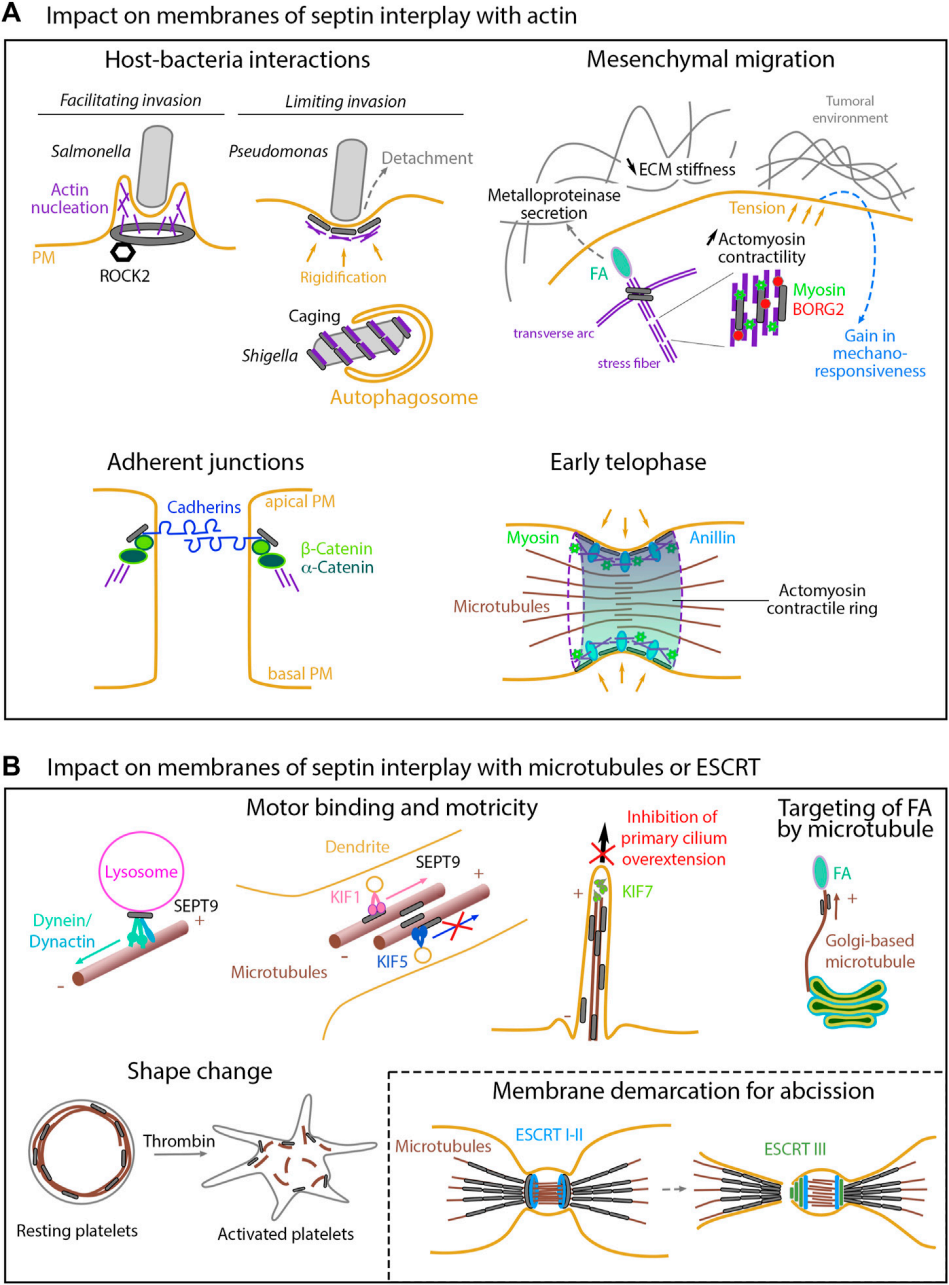
Impact on membranes of septin interplay with other cytoskeletons **(A)** Actin-related impacts. While facilitating cell invasion of specific bacteria, septins can also limit invasion either by enhancing host cell rigidity or by favoring autophagy-mediated degradation once bacteria have been internalized. During mesenchymal migration, septins contribute to the stabilization of nascent focal adhesions and to extracellular matrix degradation through metalloproteinase secretion. Septin-mediated enhancement of actomyosin contractility ultimately increase cell tension forces, thereby impacting the tumoral environment and modulating mechano-responsiveness. By linking the catenin complex to the actin cytoskeleton, septins favor cell-cell junctions required for the apico- basal polarity. During cytokinesis, membrane-associated septins and anillin are required to anchor the actomyosin contractile ring to the cell cortex. **(B)** Microtubule- and ESCRT-related impacts. Septins control the binding and motricity of molecular motors, thereby regulating the retrograde and anterograde transport on microtubules of proteins or vesicles, as illustrated with the clustering of lysosomes during cell adaptation to stress, the sorting of kinesin motor cargoes during entry into dendrites, or the control of cilia over-elongation. By guiding non-centrosomal Golgi-based microtubules, septins are also involved in focal adhesion disassembly in migrating cells. Delocalization of septins from microtubules impacts cell shape, as illustrated in platelets. During cytokinesis, membrane-bound septin rings demarcate the membrane sites for ESCRT III ring assembly prior to final abscission.

Here, we emphasize recent data that report a septin impact on membranes, either by direct interactions, or by their association with other cytoskeleton elements (Figures 1, 2). As the loss/manipulation of a distinct septin can profoundly impair filament/oligomer integrity *per se*, note that in some studies it remains open if the observed effects can be attributed to the septin under study or rather to septin oligomers/filaments.

## Direct influence of septins on membranes

Septin recruitment at plasma membrane (PM) but also at organelle or pathogen membranes relies on known interactions with lipids and mostly takes place at specific curved regions. Their principal role is to create membrane subdomains of defined composition and function to control membrane protrusion, fusion, fission, polarity, rigidity, traffic and signaling.

### Recruitment by curved membranes and lipids

Cell membrane curvatures range from nanometer to micrometer scales. Only a few proteins, including septins, can interact specifically with micrometric curvatures (Bridges et al., 2016), which are found mostly at the cytokinetic furrow and at the base of cell protrusions. *In vitro*, septin polymers lie flat along the hill axis of positive/convex curvatures while they bend orthogonally in negative/concave curvatures (in valleys), explaining the higher septin density on positive geometries in cells (Beber et al., 2019b). Septin binding to positively curved membranes relies on their amphipathic helix (AH) domains (Cannon et al., 2019; Lobato-Márquez et al., 2021; Woods et al., 2021), and on their interaction with phosphoinositides, especially PI(4,5)P2, which enhances septin arrangement into filaments (Zhang et al., 1999; Bertin et al., 2010; Beber et al., 2019a). Indeed, adjacent to the AH domains, human septins harbor two polybasic domains, which further enrich phosphoinositide-containing membranes with septins (Omrane et al., 2019). In addition, it was shown that phosphoinositides containing very-long-chain fatty acids could organize septins at the membrane of the *M. oryzae* fungus to mediate appressorium-dependent infection of rice cells (He et al., 2020). Reciprocally, septins may control the content in phosphoinositides, since the *Drosophila* Peanut/SEPT7 decreases the PIP5 kinase activity and the resulting PI(4,5)P2 resynthesis  (Kumari et al., 2022).

Besides phosphoinositides, septins can also bind to other anionic lipids such as cardiolipin (at *Shigella* poles) (Krokowski et al., 2018), to sterol-rich membrane domains (to contribute to the cell wall integrity response following membrane stress *in Aspergillus*) (Mela and Momany, 2021) or to LecA-activated Gb3 (globotriaosylceramide)-containing membranes (to rigidify the host membrane and prevent *Pseudomonas* invasion following its attachment to the host cell PM) (Aigal et al., 2022). Additionally, the protein Cdc42 at PM and its regulators define the recruitment site of septins to control bud emergence in fungi (Chollet et al., 2020; Zheng et al., 2022). Reciprocally, the other septin partners, which are gradually discovered (Benoit et al., 2021), are rather organized *via* the septin roles of diffusion barrier or scaffold, either at the plasma membrane, or at the membranes of the organelles.

### Septin functions at the plasma membrane

#### Membrane protrusions and invaginations

Once bound to membranes, septins can reshape them by promoting protrusions (Beber et al., 2019b). Indeed, in endothelial cells, the SEPT2 diffusive barrier that assembles at the base of podosomes facilitates their maturation (Collins et al., 2020). In fungi, membrane-bound septins define new regions of hyphal growth, by forming rings at the base of new lateral branches (Li et al., 2012; Feng et al., 2017). The phospholipid-binding Nim1 kinase Gin4, known to be essential for septin ring assembly at the bud neck (Longtine et al., 1998), is also required for hyphal growth of *C. albicans* (Au Yong et al., 2016). Also, septin filaments lie at the base of primary cilia where they control protein entry (Palander et al., 2017) and retain signaling receptors (Hu et al., 2010). SEPT9 builds a barrier around clathrin-rich patches to stimulate endocytosis and bone resorption by osteoclasts (Møller et al., 2018). Following oleate treatment, caveolin-bound SEPT11 relocates from caveolae of adipocytes to lipid droplets and bind the fatty acid chaperone FABP5 to contribute to insulin signaling and lipid accumulation (Moreno-Castellanos et al., 2017).

#### Exocytosis

Septins influence exocytic vesicle fusion with the PM (Yang et al., 2010), as evidenced for synaptic vesicles, GLUT4 storage vesicles in podocytes and lytic granules in natural killer cells**,** through interactions with the SNARE machinery (Beites et al., 1999; Tokhtaeva et al., 2015; Wasik et al., 2017; Phatarpekar et al., 2020). Also, septins of the *M. oryzae* fungus assemble the exocyst-tethering complex at the pore of the leave-invading structure (the appressorium) to allow local exocytosis and rice infection (Gupta et al., 2015). In this process, the osmo-sensor kinase Sln1 is required for septin ring organization and correct localization of the exocyst component Exo70 (Ryder et al., 2019).

#### Cell polarity and signaling

In neurons, septin-mediated polarity establishment occurs through membrane compartmentalization processes (Radler and Spiliotis, 2022). For example, septins demarcate the cortical sites of neurite retraction before mitosis to ensure their proper re-emergence location in daughter neurons, thereby maintaining polarity inheritance (Boubakar et al., 2017). Septins stabilize submembranous ankyrins at the axon initial segment for the control of neuronal polarity (Hamdan et al., 2020). Accordingly, in hematopoietic stem cells, the BORG4 protein, belonging to the Cdc42-regulated BORG family known to regulate the septin network (Joberty et al., 2001), compartmentalizes polarity proteins when complexed with SEPT7 (Kandi et al., 2021).

Concerning signaling, the relocalization of SEPT7 following its phosphorylation, from the base of the protrusion to the spine head membranes, restricts PSD95 protein motility to promote dendritic spine maturation (Yadav et al., 2017; Byeon et al., 2022). In astrocytes, septins interact with the glial glutamate transporter GLT-1 to reduce its mobility, and together with Cdc42EP4/BORG4 form patches that influence synaptic activity (Piniella et al., 2018). More broadly, by controlling membrane receptor localization, septins are involved in the regulation of synaptic vesicle trafficking and neurotransmitter release (Ageta-Ishihara et al., 2015; Marttinen et al., 2015). Generally, septins control signaling in diverse cell types by regulating the degradation of PM receptors, such as receptor tyrosine kinases (Vagin and Beenhouwer, 2016).

### Septin functions at subcellular organelles

#### Organelle biogenesis

SEPT7 association with fat storage-inducing transmembrane 2 protein (FIT2) at the endoplasmic reticulum (ER) is essential for lipid droplet biogenesis (Chen et al., 2021). Autophagosome formation in budding yeast involves septin ring-like structure formation at pre-autophagosomal structures and at the membrane of mature autophagosomes *via* Atg8 and Atg9 (Barve et al., 2018a; 2018b). More precisely, in neurons, Atg8/LC3B binds SEPT3 to promote autophagosome biogenesis, while SEPT5 acts as a negative regulator (Marttinen et al., 2020; Ferreira et al., 2022; Tóth et al., 2022).

#### Organelle fusion and fission

Septins can localize to mitochondria fission sites, where they bind to Drp1 and promote its recruitment to enhance mitokinesis, both in mammals and in *C. elegans* (Pagliuso et al., 2016; Sirianni et al., 2016). SEPT9 also regulates lysosome localization before their fusion with lipid droplets (Song et al., 2022). Similarly, septins are required for the fusion of macropinosomes with endosomes/lysosomes (Dolat and Spiliotis, 2016) and of entrapped *Shigella* with lysosomes (Krokowski et al., 2018).

#### Partitioning at the ER

In budding yeast, septins confine proteotoxic misfolded proteins upon ER stress in the mother compartment during cell division (Clay et al., 2014). Similarly, the septin Shs1 creates an ER barrier at the bud neck to confine the spindle capture protein Num1 to the mother cortical ER (Chao et al., 2014). More generally, as they partition the cortical ER-PM contacts, septins restrict the flow of peripheral proteins between the mother and daughter cells (Sugiyama and Tanaka, 2019). In mammals, SEPT4 regulates the number of ER-PM junctions and locally enhances the interactions between the ER Ca^2+^ sensor STromal Interaction Molecule 1 (STIM1) and the PM Ca^2+^ channel Orai1 (Katz et al., 2019; De Souza et al., 2021). By contrast, SEPT7 negatively regulates extracellular Ca^2+^ entry in neuronal ER at ER-PM contacts, by preventing Orai1/STIM1 colocalization (Deb et al., 2020; Serwach and Gruszczynska-Biegala, 2020). Also, in mouse Purkinje neurons, STIM1 deficiency can be rescued by SEPT7 loss (Dhanya and Hasan, 2021).

## Impact of septin/actin crosstalk on membrane organization and functions

Septins often coalign with actin stress fibers with which they interact either directly (Smith et al., 2015) or *via* actin binding proteins (Spiliotis, 2018). They regulate actin polymerization, stability and bundling, and their depletion or relocalization triggers the thinning of actin stress fibers (Kinoshita et al., 2002; Schmidt and Nichols, 2004; Salameh et al., 2021; Kuzmić et al., 2022), with expected impact on PM remodeling, focal adhesion (FA) turnover, cell migration, and actomyosin contractility (Lam and Calvo, 2019; Spiliotis and Nakos, 2021).

### Control of cell shape and cortical rigidity

While septins are excluded from the actin meshwork in PM ruffles, their presence at specific membrane domains stimulates actin bundling and stabilization (Mavrakis et al., 2014; Smith et al., 2015; Iv et al., 2021), increasing cell rigidity (Gilden and Krummel, 2010). During *Drosophila* wing morphogenesis, septin-mediated actin bundling is required to build up compact microtubule-actin cables into PM projections to reinforce cell-matrix adhesion (Sun et al., 2021). Septin-enhanced PM rigidity also occurs during cell rounding at the transition between interphase and mitosis (Vadnjal et al., 2022). Also, PI(4,5)P2 and the actin-binding protein anillin control septin recruitment to the periaxonal oligodendrocyte membranes to organize myelin sheaths and ensure normal nerve conduction (Patzig et al., 2016; Erwig et al., 2019).

### Pathogen invasion

Septin-mediated control of cortical PM rigidity is also used by host cells to prevent infection by microbial agents (Torraca and Mostowy, 2016; Aigal et al., 2022). If internalization occurs, septins cage bacteria or their actin tail to target them to autophagic degradation (Torraca and Mostowy, 2016; Robertin and Mostowy, 2020). During poxvirus infection, septins entrap the virus at the PM, an antiviral function regulated by actin polymerization (Ngo and Mostowy, 2019). In contrast, septin-mediated activation of actin nucleation is required for efficient *Salmonella* invasion (Boddy et al., 2018). During rice blast disease, the septin ring at the appressorium pore (Eseola et al., 2021) scaffolds actin and provides proper curvature and cortical rigidity necessary for plant cell invasion (Dagdas et al., 2012; Ryder et al., 2022).

### Control of migration

Septins regulate both mesenchymal/lamellipodial and amoeboidal/blebbing migration modes. In the lamella, septins localize at the interface of dorsal and transverse actin arcs and stabilize nascent FAs at the PM (Dolat et al., 2014). SEPT9_i1 promotes FA turnover by targeting Rho/ROCK and FAK signaling (Zeng et al., 2019), and long SEPT9_i1-3 isoforms favor metalloprotease secretion at FAs to promote extracellular matrix degradation (Marcus et al., 2019). By contrast, SEPT7 and SEPT9_i2 inhibit cell migration through actin filament disassembly (Hou et al., 2016; Verdier-Pinard et al., 2017). During amoeboid migration, septin-mediated actomyosin contractility controls PM retraction (Gilden et al., 2012), cortical rigidity, directionality (Tooley et al., 2009), and attributable to SEPT9, outside of the filamentous context, invasion and metastasis (Farrugia et al., 2020).

In cancer-associated fibroblasts, the Cdc42EP3/BORG2-dependent dense actomyosin and septin network allows high contractility and increased mechano-responsiveness to changes in extracellular matrix stiffness (Calvo et al., 2015). Regarding mechanotransduction, upregulated SEPT6 drives hepatocellular carcinoma cell proliferation, migration and invasion *via* the actin/Hippo/YAP-signaling pathway (Fan et al., 2021).

### Cytokinesis achievement

During cytokinesis, the rearrangement of septin filaments at the PM from an actomyosin-associated structure into an anillin-stabilized double ring is required for the cortical compartmentalization of cytokinetic factors, maturation of the intercellular bridge (ICB), and actomyosin constriction. Cytokinetic progression is probably the process for which the molecular mechanisms underlying the spatiotemporal septin functions and remodeling into higher-order structures are the best known. (Menon and Gaestel, 2015; Marquardt et al., 2019, 2020, 2021; Carim et al., 2020; Chen et al., 2020; Piatti, 2020; Russo and Krauss, 2021). Septin compaction into two rings is mediated by anillin (Chen et al., 2020; Arbizzani et al., 2022), a known adaptor that recruits septins on F-actin (Kinoshita et al., 2002) and on PI(4,5)P2 (Liu et al., 2012). Also, the ICB assembly relies on the recognition of specific septin units by the CIN85/anillin adaptor complex (Panagiotou et al., 2022). Furthermore, increasing tissue stiffness to mimic a tumoral environment correlates with higher expression of both anillin and SEPT6, leading to abscission failure in cells undergoing epithelial to mesenchymal transition (Simi et al., 2018; Rabie et al., 2021). Besides, the Hof1-septin scaffold directly binds and organizes actin cable spacing to ensure yeast cytokinesis (Garabedian et al., 2020).

To ensure actin contractility during division, mammalian SEPT2 scaffolds myosin II and its kinases (Joo et al., 2007). SEPT9 colocalizes with actomyosin to generate the site of membrane abscission and stabilizes the PM curvature (Wang et al., 2019). Eventually, cytokinesis completion requires SEPT6, SEPT7 and SEPT11 SUMOylation to keep their colocalization with actin and to prevent abnormal septin-bundling in the ICB (Ribet et al., 2017). Moreover, during embryogenesis, actomyosin-anchored septins are required for anaphase chiral cortical rotation in *C. elegans* zygote (Zaatri et al., 2021) and for generating submembranous curved and packed actomyosin networks during fly blastoderm cellularization, a specialized form of cytokinesis (Mavrakis et al., 2014; Xue and Sokac, 2016).

### Cell junction integrity

Septins are also important at cell junctions. During epithelial cyst morphogenesis, they localize to the lateral PM, where they link E-cadherin to beta-catenin, recruit actin filaments and favor adhesion junction and apico-basal polarity (Wang et al., 2021). In endothelial monolayers, localization of SEPT2 at the PM is required for the integrity of adherens and tight junctions to build the vascular barrier (Kim and Cooper, 2018, 2021). SEPT9 also interacts with the atypical cadherin DCHS1 to direct filamentous actin organization and cell-to-extracellular alignment in the cardiac valve (Moore et al., 2022).

## Impact of septin/microtubule interplay on membrane organization and functions

In addition to membranes and actin, septins can also bind to microtubules and to a variety of microtubule-associated proteins to control transport, cell shape, growth or adhesion (Bai et al., 2013; Spiliotis, 2018; Spiliotis and Kesisova, 2021; Spiliotis and Nakos, 2021).

### Control of cell shape, adhesion and migration

In *Xenopus* embryos, SEPT7-mediated reorientation of microtubules along the PM is required for cell elongation during wound closure (Shindo et al., 2018). Strikingly, SEPT9 is one of the five proteins that mediate crosstalk between actin, FA and microtubules (Paradžik et al., 2022). By analogy, the *C. difficile* bacteria, which produce a toxin that locally depolymerizes cellular actin, cause septin-dependent formation of microtubule-based protrusions that enhance bacterial adhesion (Nölke et al., 2016). At the Golgi, PI4P recruits SEPT9 to perform Golgi clustering (Omrane et al., 2019), while GM130/GOLGA2 recruits SEPT1, which, independently of filaments, performs local microtubule nucleation and stimulates anterograde membrane traffic (Song et al., 2019). Additionally, FA-proximal septin filaments promote Golgi-based non-centrosomal microtubule growth into FAs, thereby regulating FA turnover and cell migration (Merenich et al., 2022).

### Regulation of molecular motor binding

In RPE1 primary cilium, septins are required to accumulate the microtubule-capping kinesin KIF7 at the tip, which prevents cilia overextension (Kanamaru et al., 2022). Recent data highlight the control that septins exert over the binding of molecular motors to cargoes or to microtubules. They act as scaffolds for the binding of dynein-dynactin to lysosomes in retrograde transport (Kesisova et al., 2021). Septins also control the entry of cargoes into dendrites *via* regulating their loading on KIF17 (Bai et al., 2016). In line with this, microtubule-bound SEPT9 specifically prevents KIF5-mediated centrifugal transport of membrane proteins, while it boosts the KIF1-mediated one (Karasmanis et al., 2018). SEPT7 binds to the kinesin KIF20A/MKLP2 in the ICB of dividing neuronal progenitors, to maintain the proliferative state of these cells, which is critical for proper brain development (Qiu et al., 2020). Recently, a hypothetical septin code has been proposed to explain how specific septin polymers differentially recruit kinesin and dynein motors to regulate traffic along microtubule tracks (Spiliotis and Kesisova, 2021).

### Impact of subcellular relocalization

Actin-to-microtubule relocalization of SEPT9 following forchlorfenuron/UR214-9 treatment enhances kinesin-microtubule interactions and microtubule bundling into a subcortical giant ring, resulting in a discoid cell architecture in breast cancer cells (Zhovmer et al., 2021). Such a septin-associated microtubule ring is naturally present in resting platelets but dissociates upon their activation (Kim et al., 2022). Accordingly, the redirection of SEPT4 and 8 to the cortex following activation suggests a functional role of at least both septins in platelet granular secretion (Bläser et al., 2004). Moreover, patients bearing a SEPT9 congenital variant suffer from granule secretion defect (Neubauer and Zieger, 2021). Otherwise, octamers that include SEPT9_i1 can relocalize from F-actin to microtubules, which results in a relaxation of peripheral actin stress fibers, affecting actomyosin organization and tension (Kuzmić et al., 2022). Such septin relocalization induces acquired resistance to the microtubule-stabilizing chemotherapy drug paclitaxel (Targa et al., 2019; Salameh et al., 2021).

## Impact of septin interplay with ESCRT or intermediate filaments on membranes

During cytokinesis, septins rearrange into two membrane-bound septin rings, which demarcate the membrane sites for ESCRT-III ring assembly into functional cones for final abscission. Indeed, the ESCRT-III recruitment occurs concomitantly with the disassembly of the septin double ring (Addi et al., 2018; Karasmanis et al., 2019; Russo and Krauss, 2021). In non-adherent cells, where ESCRT rings do not assemble, cytokinesis fails because of ICB regression. However, in case of re-adhesion, SEPT7 stabilizes the ingressed PM, thereby restoring tension-mediated rupture in an ESCRT-independent way (Gupta et al., 2018).

At last, intermediate filaments (IFs) have also been identified as binding partners of oncogenic SEPT9 isoforms by proteomic profiling (Devlin et al., 2021). During human spermiogenesis, SEPT12 is recovered in a complex with the IF nucleo-cytoskeleton laminB1 and the nuclear membrane cytoskeleton-linker protein SUN4/SPAG4 during the formation of nuclear envelopes of round spermatids (Yeh et al., 2015). Also, SEPT12 overexpression acts independently of filaments to disrupt laminA/C distribution and sperm morphogenesis (Yeh et al., 2019).

## Conclusion

Even though the underlying mechanisms have most often not been elucidated, the direct and indirect roles of septins in membrane organization and functions keep growing, with clear indications that cytoskeleton interplays are key in these processes. Given the pivotal roles of septin filaments in fine-tuning local membrane properties, it seems obvious that their deregulations will be increasingly highlighted in various pathologies, including cancers, neuropathies, infections, ciliopathies, obesity or male sterility.
